# Effects of photoperiod on performance, ovarian morphology, reproductive hormone level, and hormone receptor mRNA expression in laying ducks

**DOI:** 10.1016/j.psj.2021.01.002

**Published:** 2021-01-22

**Authors:** Yao-ming Cui, Jing Wang, Hai-jun Zhang, Guang-hai Qi, Shu-geng Wu

**Affiliations:** ∗ Laboratory of Quality & Safety Risk Assessment for Animal Products on Feed Hazards (Beijing) of the Ministry of Agriculture and Rural Affairs Feed Research Institute, Chinese Academy of Agricultural Sciences, Beijing 100081, China; † College of Biological Engineering, Henan University of Technology, Zhengzhou, Henan province 450000, China

**Keywords:** follicle, laying duck, ovarian morphology, photoperiod, reproductive hormone

## Abstract

We investigated the effect of photoperiod on performance, ovarian morphology, reproductive hormones levels, and their receptors mRNA expressions in laying ducks. After adaption, 300 252-day-old Jinding laying ducks were randomly allocated to 5 groups, receiving 12L:12D, 14L:10D, 16L:8D, 18L:6D, or 20L:4D, respectively. Each treatment had 6 replicates of 10 birds each. The feeding trial lasted 8 wk. Egg production, egg mass, and ADFI increased linearly and quadratically with increasing photoperiods (*P* < 0.05), and the higher values of them occurred in photoperiods ≥ 16 h, compared with 12L:12D (*P* > 0.05). Initial and bare stroma weight increased quadratically, while total large white follicle (**LWF**) number and weight increased linearly and quadratically, with increasing photoperiods (*P* < 0.05). The higher values of them occurred in 16L:8D and 18L:6D treatments as well as the higher total LWF weight also occurred in 20L:4D, compared with 12L:12D (*P* > 0.05). Besides, 16.93 and 16.93 h were the optimal photoperiods for bare stroma (follicles ≥ 2 mm in diameter removed) weight and total LWF weight, respectively, calculated from reliable regression equations (*R*^*2*^ ≥ 0.5071). Compared with 12L:12D, the higher levels of estradiol, progesterone, follicle-stimulating hormone (**FSH**) as well as the higher expressions of estrogen, luteinizing hormone (**LH**) and progesterone receptors were observed in ≥16 h photoperiods (*P* < 0.05), while the higher LH level and FSH receptor expression only occurred in 16L:8D and 18L:6D (*P* < 0.05). In the hypothalamus, higher mRNA expression of gonadotropin-releasing hormone occurred in 16L:8D and 18L:6D groups (*P* < 0.05). Meanwhile, gonadotropin-inhibitory hormone and prolactin increased in 20-hour photoperiod (*P* < 0.05), and the latter may be due to theup-regulation of vasoactive intestinal peptide expression (*P* < 0.05). To sum up, an appropriate photoperiod could improve the performance and reproductive organ and ovarian follicles development through reproductive hormones and their receptors, and 16.56 to 10.93 h is an adequate photoperiod for laying ducks.

## Introduction

Laying duck production is an enormous industry in China, producing 3,070 thousand eggs in 2018, which mean 42.3 billion yuan (Liu and Xu, 2019). The productive performance of laying bird is closely related with the development of reproductive organs and ovarian follicles (Liu and Zhang, 2008; Long et al., 2017). In fact, the course of ovarian follicles development follows a rigorous hierarchical system which is the result of atresia-related mechanism (Lei et al., 2014). Concretely, the third-largest follicle (**F3**) will become the new largest follicle (**F1**) or the second-largest follicle (**F2**), following the maturation and ovulation of F1 and F2. Meanwhile, a small yellow follicle (**SYF**) will be chosen to enter the preovulatory follicle (**POF**) hierarchy. In poultry, photoperiod is one of the most significant environmental factors which impact reproductive activity, through regulating the development of reproductive organ and ovarian follicle (Chen et al., 2007; Hassan et al., 2013).

The productive activity, reproductive organs, and ovarian follicle development of laying birds are mainly regulated by reproductive hormones in hypothalamic-pituitary-gonad (**HPG**) axis (Hernandez and Bahr, 2003; Hassan et al., 2013). The upregulation of reproductive hormone receptor expression could be ascribed to the increases in secretion and release of corresponding hormone (Yin et al., 2018), while the latter could be affected by the upstream hypothalamic gonadotropin-releasing hormone (**GnRH**) neurons activity. In fact, photoperiod was a crucial modulator for GnRH neurons activity (Sharp, 1993). In fowl, the secretion and release of GnRH could be regulated by deiodinase type 2 and 3 (**Dio2/Dio3** system) (Perfito et al., 2015) and be inhibited by gonadotropin-inhibitory hormone (**GnIH**) (Banerjee and Chaturvedi, 2017) or prolactin (Sharp and Blache, 2003). However, to the best of our knowledge, the influences of photoperiod (from 12–20 h) on Dio2/Dio3 system, GnIH, and prolactin in birds are minimally reported and even have not been seen in laying ducks. Therefore, the effects of photoperiod on Dio2/Dio3 system, GnIH, and prolactin needs to be investigated, to explore the pathway in which photoperiod impacts the secretion and release of reproductive hormone of laying ducks.

There has been no consistent photoperiod protocol for laying ducks in practical production. Hence, more work is needed to evaluate the effect of photoperiod on the productive performance, reproductive organ and ovarian follicle development, and further explore the optimal photoperiod for laying ducks. Moreover, although the functions of these hormones have been extensively reported (Nakada et al., 1994; Hernandez and Bahr, 2003), the influences of photoperiod on the synthesis, secretion, and release of them as well as their receptors' gene mRNA expression remain unclear for laying birds. Therefore, more work is required to investigate how different photoperiods impact the levels of reproductive hormones in serum and the mRNA expression of their receptors in target tissues of laying birds. Jinding laying duck is a typical native breed, with the mean egg production of 75.15 ± 3.29% and mean BW of 1.66 ± 0.16 kg (Gao et al., 2010). The age at the first egg of Jinding laying ducks is about 140 d (Cui et al., 2019a), and the laying period can last for more than 1.5 yr. The purpose of this research was to evaluate the effect of photoperiod on productive performance, reproductive organ and ovarian follicle development, reproductive hormone levels in serum, their receptors mRNA expression in ovarian follicles, as well as GnRH expression and regulation. An appropriate photoperiod for Jinding laying ducks was expected to obtain.

## Materials and methods

### Birds, Treatments, and Husbandry

All experimental protocols were approved by the Animal Care and Use Committee of the Feed Research Institute of the Chinese Academy of Agricultural Sciences. After adaption, 300252-day-old Jinding laying ducks were randomly allocated to 5 treatments with a corn–soybean meal diet (Table 1) for 8 wk. Each treatment had 6 replicates of 10 ducks per replicate. Every replicate was raised in an individual room (200 × 90 × 60 cm; length × width × height) containing automatically controlled light timers as well as adjustable light intensity, temperature, and ventilation (Cui et al., 2019a). Birds received 5 lighting programs: 12L:12D, 14L:10D, 16L:8D, 18L:6D, and 20L:4D, respectively. During the light hour, all ducks received light-emitting diode light with an average intensity of 20 (±1.0) lux at eye level. All the lighting programs were artificial. The previous lighting program before the experimental period was natural light (about 12 h of light). Air quality was guaranteed by a programmed ventilation of the whole aviary combined with top–down ventilation of individual room and cleaning of litters twice a day. Diet (in pellet form) and water were provided ad libitum, and feed intake was limited every day.

**Table 1:** Composition and nutrient content of diet for laying ducks (air-dry basis, %).

Items	Contents (%)
Ingredient	
Corn	60.60
Soybean meal	27.60
Salt	0.30
Calcium phosphate	1.00
Limestone	8.50
DL-methionine	0.23
Choline chloride	0.10
Lys-HCl	0.10
Premix^1^	0.57
Oil	1.00
Nutrient level^2^	
AME (MJ/kg)	11.25
CP	17.00 (17.28)
Calcium	3.15 (3.12)
Total phosphorus	0.56 (0.57)
Available phosphorus	0.34
Lysine	0.92
Methionine + cystine	0.74

1 Premix supplied per kg of diet: vitamin A, 12,500 IU; vitamin D_3,_ 4,125 IU; vitamin E, 15 IU; vitamin K_3,_ 2 mg; thiamine, 1 mg; riboflavin, 8.5 mg; pyridoxine, 8 mg; vitamin B_12,_ 5 mg; biotin, 2 mg; folic acid, 5 mg; Ca-pantothenate, 50 mg; niacin, 32.5 mg; Cu, 10 mg; Zn, 72 mg; Fe, 59 mg; Mn, 57 mg; Se, 0.15 mg; I, 0.50 mg.

2 The values in parentheses indicate the analyzed values. Others are calculated values.

### Performance

Before the formal experiment, the egg production of all replicates was adjusted to be approximately equal. Eggs were collected, and irregular (misshapen, broken, and soft) eggs were noted daily. Egg production, egg weight, and mortality were recorded every day by replicate. Feed consumption per replicate was weighted and recorded every 4 wk. Egg mass was calculated by multiplying average egg weight by egg production. Feed conversion ratio was obtained as grams of total feed consumption/total egg weight per replicate. ADFI and feed conversion ratio were calculated by replicate each 4 wk. Egg production and ADFI were adjusted by duck mortality in time.

### Hypothalamus and Reproductive Organ Sampling

At the end of the experiment (310 d of age), 12 ducks from each group were randomly chosen (2 birds per replicate) and quickly killed by an overdose of anesthesia (pentobarbital sodium). Hypothalamus samples were collected, immersed in liquid nitrogen, and then stored at −80°C for subsequent measurement of mRNA expression for genes encoding GnRH, GnIH, Dio2, Dio3, and vasoactive intestinal peptide. In addition, the oviduct and ovary were collected and weighed. The initial stroma was weighted after the POF (>10 mm in diameter) being removed from the ovary, and the bare stroma was weighted after the SYF (6–10 mm in diameter) and LWF (2–5 mm in diameter) being removed from the initial stroma.

### Collection of Ovarian Follicles

The preovulatory follicles, including F1, F2, F3, SYF, and LWF, were removed from the ovary, counted, weighed, and noted. Then, the granulosa layers of F1, F2, and F3 were divided, as per the description of Gilbert et al. (1977). The granulosa layer, SYF, and LWF with average sizes were immersed in liquid nitrogen and stored at −80°C for the mRNA expression measurement (Long et al., 2017) of the genes which encode follicle-stimulating estrogen receptor (**ER**), hormone receptor (**FSHR**), luteinizing hormone receptor (**LHR**), and progesterone receptor (**PR**).

### Serum Hormone Analysis

At the end of 309 d of age, 2 birds from each replicate were fasted for 12 h, and then, ∼3 mL blood was obtained from a wing vein of duck using evacuated tubes with coagulant. Blood collection was limited in 1 h, from 0:00, 6:00, 12:00 and 18:00, respectively. Serum samples were obtained as per the method reported by Cui et al. (2019b), and then stored at ‒20°C for the following analysis. After being thawed at 4°C overnight, the levels of prolactin, estradiol, follicle-stimulating hormone (**FSH**), luteinizing hormone (**LH**), and progesterone were measured using ELISA kits for ducks (Nanjing Jiancheng, Bioengineering Institute, Jiangsu, China; Xu et al., 2009), with horse radish peroxidase marking the second antibody and tetramethylbenzidine serving as a chromogenic reagent.

### Quantification of Reproductive Hormone Receptor mRNA with Real-Time PCR

Total RNA was obtained from granulosa layers, SYF, LWF, and hypothalamus samples using TRIzol reagent (Tiangen Biotech Co., Ltd., Beijing, China). The yield and integrity of RNA were determined using a NanoDrop 2000 spectrophotometer (Thermo Fisher Scientific, Waltham, MA), and agarose−ethidium bromide electrophoresis. Expression quantification was conducted with a two-step reaction process, which contains reverse transcription and PCR using FastQuant Reverse Transcription kit (KR106; Tiangen, Beijing, China), as per the description of Cui et al. (2019b). The relative mRNA expressions were normalized to avian β-actin with the 2^−ΔΔCt^ method (Livak and Schmittgen, 2001). Primer sequences are detailed in Table 2.

**Table 2:** Primer sequence of target and reference genes.

Gene	Forward primer (5′-3′)	Reverse primer (3′-5′)	GenBank number	Length (bp)
Estrogen receptor	TGGTGGGTTTGATGTGGAG	TCTTCTTGGACTTTCGTTGTC	EU014164.1	247^1^
Progesterone receptor	ATGGTCCTGGGAGGTCGAAA	ACTTCTGGCTCAATGCCTCG	XM_027467850.1	197
Follicle-stimulating hormone receptor	GGAACATACCTGGATGAGCTA	GTCCAGTGCCTAATCTTGAG	EU049608.1	147^1^
Luteinizing hormone receptor	ACTTGCGTATGACAACCATACC	CTCAGGGACGGATCAATGCC	EU049613.1	250^1^
Gonadotropin-releasing hormone	ACACTGGTCTTATGGCCTGC	TAAGAGCCAGGGCATTCAGC	NM_001080877.1	129
Gonadotropin-inhibitory hormone	TCAAGGCGTCCAGGAATCTG	TCTGGGTCTTTCGGTTTCCA	XM_015853673.1	92
Deiodinase type 2	ACAAGCAGGTCAAATTGGGAG	GGCAAAATCCAGAAGGTGGC	XM_013094234.3	137
Deiodinase type 3	CCTACGGTGCCTACTTCGAG	CTCTGGAGCCGGGTTTTGTA	XM_005031806.3	136
Vasoactive intestinal peptide	AGTCCTGTCAAACGCCACTC	TTCCTGGCTTCTTTTTCCGGT	XM_027454372.1	117
β-actin	ATGTCGCCCTGGATTTCGAG	CATGGGCCCGTAGCGACTGT	EF667345.1	282^1^

1 Sequences based on Song et al. (2009).

### Statistical Analysis

All analyses were performed using SAS, version 9.2,(2001, SAS Institute, NC). The replicate (1 room per replicate) was the experimental unit for the analysis of performance. For the other parameters, the mean of 2 ducks serving as the experimental unit for statistical analysis. The homogeneity of variances and normality of the data were evaluated first, which are the preconditions of ANOVA (Cui et al., 2019a). The Shapiro-Wilk test was adopted to investigate normality. Then, 1-way ANOVA and Duncan multiple range test were used for data analysis. Regression analysis was used to evaluate the linear and quadratic effects of photoperiod. Arcsine transformation was carried out before egg production data statistical analysis (Cui et al., 2019c). Differences were supposed to be statistically significant at *P* < 0.05. Data were showed as the mean and pooled SEM.

The PROC REG used in regression analysis and statistical models were as follows (Cui et al., 2018), Yij = α + β_1_Xi + eij (linear regression), Yij = α + β_1_Xi + β_2_Xi^2^ + eij (quadratic regression).

Yij was the response variable; α was the intercept (indicators with the 12 h of light); β_1_ and β_2_ were regression coefficient; Xi was the studied factor effect as hour of light (i = 12, 14, 16, 18, 20), and eij was the observational error for (ij)th observation.

## Results

### Performance

The effect of photoperiod on performance of laying ducks is presented in Table 3. No significant differences in egg production were observed among all the treatments at the beginning of the experiment (*P* > 0.05, data not shown). Egg weight and feed conversion ratio were not affected by photoperiod during the whole period (*P* > 0.05). Increment in photoperiod linearly and quadratically increased egg production, egg mass, and ADFI (*P* < 0.05), during 1–4, 5–8 and 1–8 wk of the experiment. Compared with 12L:12D, the higher values of egg production, egg mass, and ADFI occurred in ≥16-hour photoperiods (*P* < 0.05), during 1–4, 5–8, and 1–8 wk of the trial.

**Table 3:** Effect of photoperiod on performance of laying ducks from 37 to 44 wk of age.^1^

Items	Photoperiod					SEM	*P*-value		
	12L:12D	14L:10D	16L:8D	18L:6D	20L:4D		ANOVA	Linear^2^	Quadratic^2^
Egg production (%)									
1–4 wk	64.29^b^	66.64^b^	72.28^a^	71.67^a^	72.41^a^	0.91	0.003	<0.001	<0.001
5–8 wk	64.94^b^	67.66^a,b^	72.40^a^	71.90^a^	72.12^a^	0.86	0.008	0.001	0.001
1–8 wk	64.61^b^	67.13^a,b^	72.34^a^	71.79^a^	72.27^a^	0.87	0.004	<0.001	<0.001
Egg weight (g)									
1–4 wk	74.22	75.23	74.22	74.46	73.87	0.50	0.94	0.68	0.83
5–8 wk	73.63	74.45	74.78	74.44	73.84	0.52	0.96	0.91	0.73
1–8 wk	73.91	74.83	74.50	74.44	73.85	0.45	0.96	0.87	0.77
Egg mass (g)									
1–4 wk	47.64^b^	50.13^a,b^	53.66^a^	53.40^a^	53.46^a^	0.73	0.017	0.002	0.003
5–8 wk	47.69^c^	50.30^b,c^	54.17^a^	53.51^a,b^	53.22^a,b^	0.65	0.002	<0.001	<0.001
1–8 wk	47.67^b^	50.20^a,b^	53.92^a^	53.46^a^	53.34^a^	0.67	0.004	0.001	<0.001
ADFI (g/hen per day)									
1–4 wk	159^b^	164^a,b^	168^a^	169^a^	171^a^	1.32	0.009	<0.001	<0.001
5–8 wk	159^b^	165^a,b^	172^a^	170^a^	169^a^	1.42	0.020	0.004	0.012
1–8 wk	159^b^	164^a,b^	170^a^	170^a^	170^a^	1.17	0.002	<0.001	<0.001
Feed conversion ratio (feed/egg, g/g)									
1–4 wk	3.35	3.28	3.15	3.19	3.21	0.05	0.78	0.32	0.45
5–8 wk	3.35	3.28	3.20	3.19	3.17	0.03	0.49	0.078	0.18
1–8 wk	3.35	3.28	3.17	3.19	3.19	0.04	0.60	0.15	0.26

^a–c^Values within a row with no common superscripts differ significantly (*P* < 0.05).

1 Data are the mean of 6 replicates with 10 birds each.

2 Linear and quadratic effects of photoperiod were evaluated using regression analysis.

### Reproductive Organ Development

As shown in Table 4, no significant differences were observed in oviduct weight and percentage, ovary weight and percentage, and initial stroma percentage among all the treatments (*P* > 0.05). Initial stroma weight, bare stroma weight, and percentage were quadratically affected in response to the increasing photoperiods (*P* < 0.05). Compared with 12L:12D, the higher initial and bare stroma weight occurred in 16L:8D and 18L:6D treatments (*P* < 0.05), while the higher bare stroma percentage occurred in 16L:8D (*P* < 0.05).

**Table 4:** Effect of photoperiod on reproductive organ of laying ducks (310 d of age).^1^

Items^2^	Photoperiod					SEM	*P*-value		
	12L:12D	14L:10D	16L:8D	18L:6D	20L:4D		ANOVA	Linear^3^	Quadratic^3^
Oviduct weight (g)	45.50	49.07	50.96	49.73	51.69	1.31	0.64	0.16	0.33
Oviduct percentage (%)	2.86	2.95	3.09	2.94	3.11	0.06	0.69	0.26	0.52
Ovary weight (g)	57.15	58.56	65.62	60.80	64.62	2.03	0.65	0.24	0.46
Ovary percentage (%)	3.58	3.51	3.97	3.64	3.90	0.11	0.65	0.35	0.64
Initial stroma weight (g)	7.53^c^	7.96^b,c^	8.85^a^	8.47^a,b^	7.87^b,c^	0.12	<0.001	0.17	<0.001
Initial stroma percentage (%)	0.48	0.48	0.54	0.50	0.48	0.008	0.061	0.60	0.065
Bare stroma weight (g)	4.24^b^	4.39^b^	4.85^a^	4.69^a^	4.39^b^	0.05	<0.001	0.099	<0.001
Bare stroma percentage (%)	0.27^b^	0.26^b^	0.29^a^	0.28^a,b^	0.27^b^	0.003	0.002	0.50	0.019

^a-c^Values within a row with no common superscripts differ significantly (*P* < 0.05).

1 Data are the mean of 6 replicates with 2 birds each.

2 Initial stroma = ovary without the preovulatory follicles (follicles > 10 mm diameter removed); Bare stroma = initial stroma without the small yellow follicles or large white follicles (follicles ≥ 2 mm diameter removed).

3 Linear and quadratic effects of photoperiod were evaluated using regression analysis.

### Ovarian Follicle Development

The effect of photoperiod on the development of ovarian follicles, including F1, F2, F3, other POF, SYF, and LWF, is shown in Table 5. There were no significant differences in the weight of F1, F2, F3, total POF, mean POF, total SYF, mean SYF and mean LWF, and the number of POF, SYF, and atretic follicles, among all the groups (*P* ˃ 0.05). Number of LWF and total LWF weight increased lineally and quadratically in response to the increasing photoperiods (*P* < 0.05). Compared with 12L:12D, the higher total LWF weight was observed in ≥16-hour photoperiods (*P* < 0.05), while the larger number of LWF occurred in 16L:8D and 18L:6D treatments (*P* < 0.05).

**Table 5:** Effect of photoperiod on ovarian follicle of laying ducks (310 d of age).^1^

Items	Photoperiod					SEM	*P*-value		
	12L:12D	14L:10D	16L:8D	18L:6D	20L:4D		ANOVA	Linear^2^	Quadratic^2^
F1 weight (g)	18.67	19.20	20.50	19.52	21.41	0.49	0.44	0.097	0.26
F2 weight (g)	14.74	15.02	17.05	15.21	16.38	0.60	0.73	0.42	0.67
F3 weight (g)	8.98	9.21	10.30	8.74	10.08	0.46	0.80	0.60	0.87
Number of POF	7.17	7.33	8.00	7.67	7.83	0.43	0.98	0.59	0.84
Total POF weight (g)	49.62	50.60	56.77	52.33	56.75	2.03	0.72	0.27	0.54
Mean POF weight (g)	7.24	7.41	7.32	6.96	7.62	0.26	0.96	0.87	0.93
Number of SYF	9.83	10.17	11.67	10.67	10.33	0.50	0.84	0.68	0.62
Total SYF weight (g)	2.35	2.55	2.78	2.59	2.41	0.09	0.57	0.81	0.26
Mean SYF weight (mg)	244	253	242	249	242	4.62	0.93	0.70	0.83
Number of LWF	17.17^b^	17.67^b^	23.50^a^	23.83^a^	22.17^a,b^	0.94	0.039	0.012	0.018
Total LWF weight (g)	0.94^c^	1.02^b,c^	1.22^a^	1.19^a^	1.07^b^	0.02	<0.001	0.015	<0.001
Mean LWF weight (mg)	55.93	58.30	53.73	51.46	50.50	1.68	0.61	0.14	0.33
Atretic follicle number	3.83	3.83	3.67	3.83	4.00	0.26	0.99	0.86	0.94

^a-c^Values within a row with no common superscripts differ significantly (*P* < 0.05).

Abbreviations: F1, the first largest one of POF; F2, the second largest one of POF; F3, the third largest one of POF; LWF, large white follicles (2 to 5 mm diameter); POF, preovulatory follicle, ˃10 mm diameter; SYF, small yellow follicles (6 to 10 mm diameter).

1 Data are the mean of 6 replicates with 2 birds each.

2 Linear and quadratic effects of photoperiod were evaluated using regression analysis.

### Serum Reproductive Hormone

The changes of serum reproductive hormones (equidistant 4 time points) in response to photoperiods are detailed in Table 6. Linear and quadratic increases were observed in serum levels of estradiol (linear: 6:00 and 18:00; quadratic: 6:00, 12:00, and 18:00), FSH (linear and quadratic: 6:00), LH (linear: 6:00 and 12:00; quadratic: 6:00, 12:00, and 18:00), and progesterone (linear and quadratic: 6:00 and 18:00) in response to increasing photoperiods (*P* < 0.05). Meanwhile, the increment in photoperiod quadratically increased the levels of estradiol (12:00) and LH (18:00; *P* < 0.05). Compared with 12L:12D, the higher levels of estradiol (18:00) were observed in ≥14-hour photoperiods (*P* < 0.05), and the higher values of estradiol (6:00), FSH (6:00), and progesterone (6:00 and 18:00) occurred in ≥16-hour photoperiods (*P* < 0.05). Meanwhile, 16L:8D and 18L:6D treatments had the higher values of estradiol (12:00) and LH (12:00 and 18:00; *P* < 0.05).

**Table 6:** Effect of photoperiod on reproductive hormone levels in serum of laying ducks (309 d of age).^1^

Items	Photoperiod					SEM	*P*-value		
	12L:12D	14L:10D	16L:8D	18L:6D	20L:4D		ANOVA	Linear^2^	Quadratic^2^
Estradiol (pg/mL)									
0:00	279	297	273	302	287	6.89	0.69	0.68	0.90
6:00	409^b^	483^b^	680^a^	698^a^	706^a^	26.75	<0.001	<0.001	<0.001
12:00	854^c^	972^b,c^	1,096^a^	1,004^a,b^	935^b,c^	21.81	0.003	0.21	0.001
18:00	871^d^	989^b,c^	1,113^a^	1,090^a,b^	979^c^	22.14	<0.001	0.040	<0.001
Follicle-stimulating hormone (IU/L)									
0:00	2.99	3.27	2.95	2.91	3.06	0.09	0.78	0.75	0.95
6:00	4.90^b^	5.90^a,b^	7.21^a^	7.32^a^	7.40^a^	0.29	0.008	0.001	0.001
12:00	8.11	7.96	8.66	8.46	8.20	0.15	0.60	0.53	0.54
18:00	8.24	8.74	9.30	9.28	8.98	0.19	0.41	0.14	0.14
Luteinizing hormone (IU/L)									
0:00	3.45	3.88	3.80	3.59	3.50	0.06	0.094	0.64	0.058
6:00	3.87	4.36	4.41	4.56	4.79	0.10	0.056	0.003	0.012
12:00	6.42^b^	6.36^b^	7.58^a^	7.44^a^	7.10^a,b^	0.14	0.006	0.014	0.011
18:00	6.60^b^	7.11^a,b^	7.66^a^	7.51^a^	7.17^a,b^	0.12	0.028	0.063	0.005
Progesterone (pg/mL)									
0:00	253	272	266	245	270	7.11	0.74	0.90	0.99
6:00	332^c^	354^b,c^	400^a,b^	417^a^	415^a^	9.39	0.003	<0.001	<0.001
12:00	395	407	464	441	436	9.21	0.12	0.074	0.065
18:00	424^b^	431^b^	510^a^	477^a^	473^a^	8.12	0.001	0.010	0.003

^a-d^Values within a row with no common superscripts differ significantly (*P* < 0.05).

1 Data are the mean of 6 replicates with 2 birds each.

2 Linear and quadratic effects of photoperiod were evaluated using regression analysis.

### Reproduction-Related mRNA Expression in Follicles

Real-time PCR analyses of ER, FSHR, LHR, and PR mRNA expressions in the granulosa cell layers of POF (F1, F2, and F3), SYF, and LWF are shown in Table 7. Linear and quadratic upregulation were observed in ER (linear: F1, F2, and F3; quadratic: F1, F2, F3, and SYF), FSHR (linear and quadratic: F2, F3, and SYF), LHR (linear: F1; quadratic: F1, F2, SYF, and LWF), PR (linear and quadratic: F1, F2, F3, and LWF) in response to the increase in photoperiod (*P* < 0.05).

**Table 7:** Effect of photoperiod on the relative mRNA expressions of reproductive hormone receptor genes in laying ducks (310 d of age).^1^

Items	Photoperiod					SEM	*P*-value		
	12L:12D	14L:10D	16L:8D	18L:6D	20L:4D		ANOVA	Linear^2^	Quadratic^2^
Estrogen receptor									
F1	1.00^b^	1.05^b^	1.59^a^	1.56^a^	1.49^a^	0.07	0.004	0.002	0.002
F2	1.00^b^	1.05^b^	1.49^a^	1.47^a^	1.35^a^	0.06	0.003	0.003	0.002
F3	1.00^b^	1.08^b^	1.31^a^	1.32^a^	1.17^a,b^	0.04	0.015	0.027	0.005
SYF	1.00	1.10	1.26	1.24	1.18	0.04	0.15	0.057	0.039
LWF	1.00	1.07	1.13	1.14	1.08	0.03	0.64	0.32	0.28
Follicle-stimulating hormone receptor									
F1	1.00	1.02	1.06	1.06	1.03	0.04	0.99	0.74	0.88
F2	1.00^b^	1.13^a,b^	1.40^a^	1.39^a^	1.28^a,b^	0.05	0.042	0.018	0.010
F3	1.00^b^	1.18^b^	1.59^a^	1.57^a^	1.28^a,b^	0.06	0.005	0.032	0.002
SYF	1.00^b^	1.06^b^	1.33^a^	1.30^a^	1.17^a,b^	0.04	0.007	0.024	0.004
LWF	1.00	1.05	1.17	1.18	1.12	0.03	0.42	0.13	0.16
Luteinizing hormone receptor									
F1	0.09^b^	0.27^a,b^	0.37^a^	0.46^a^	0.36^a^	0.07	0.021	0.014	0.003
F2	0.21	0.58	0.18	0.15	0.11	0.06	0.20	0.098	0.044
F3	0.11	0.34	0.21	0.24	0.16	0.04	0.40	0.20	0.12
SYF	0.15	0.26	0.06	0.46	0.20	0.05	0.17	0.12	0.039
LWF	0.16^b^	0.15^a,b^	0.15^a^	0.11^a^	0.08^a,b^	0.03	0.018	0.093	0.004
Progesterone receptor									
F1	1.00^b^	0.98^b^	1.29^a^	1.30^a^	1.21^a^	0.04	0.003	0.004	0.005
F2	1.00	1.08	1.26	1.20	1.18	0.03	0.068	0.029	0.022
F3	1.00	1.04	1.17	1.17	1.15	0.03	0.10	0.019	0.029
SYF	1.00	1.09	1.17	1.18	1.14	0.03	0.26	0.070	0.068
LWF	1.00^b^	1.08^a,b^	1.25^a^	1.23^a^	1.22^a^	0.03	0.047	0.009	0.011

^a-b^Values within a row with no common superscripts differ significantly (*P* < 0.05).

Abbreviations: F1, the first largest one of POF; F2, the second largest one of POF; F3, the third largest one of POF; LWF, large white follicles (2 to 5 mm diameter); POF, preovulatory follicle, >10 mm diameter; SYF, small yellow follicles (6 to 10 mm diameter).

1 Data are the mean of 6 replicates with 2 birds each.

2 Linear and quadratic effects of photoperiod were evaluated using regression analysis.

Compared with 12L:12D, the higher mRNA expressions of ER (F1 and F2), LHR (F1), and PR (F1 and LWF) occurred in ≥16-hour photoperiods (*P* < 0.05); 16L:8D and 18L:6D treatments upregulated the mRNA expressions of ER (F3), FSHR (F2, F3, and SYF), and LHR (LWF; *P* < 0.05).

### Reproduction-Related mRNA Expression in the Hypothalamus and Serum Level of Prolactin

**Figure 1:**
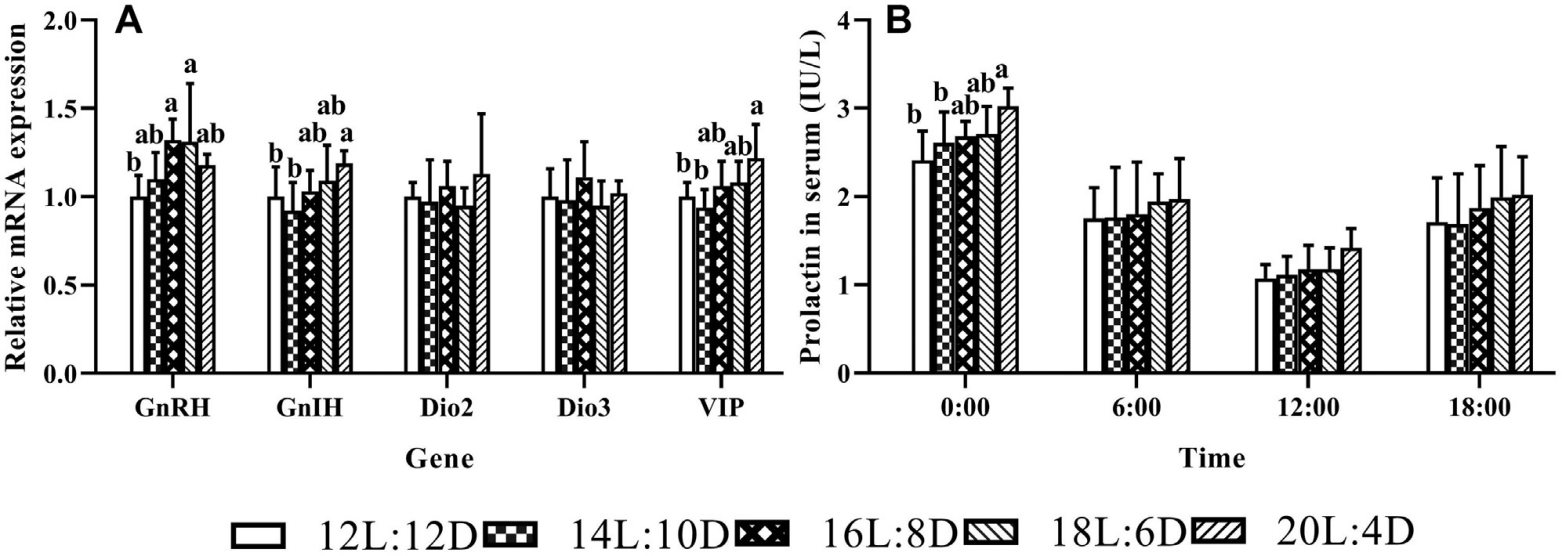
(A–B) Effect of photoperiod on the relative mRNA expressions of reproduction-related genes in the hypothalamus (310 d of age) and prolactin content in serum of laying ducks (309 d of age). The mRNA expressions were determined by quantitative real-time PCR and calculated relative to the β-actin gene. Abbreviations: Dio2, deiodinase type 2; Dio3, deiodinase type 3; GnIH, gonadotropin-inhibitory hormone; GnRH, gonadotropin-releasing hormone; VIP, vasoactive intestinal peptide. Values are expressed as relative expression ratios compared with respective controls (12L:12D). Means were calculated from 6 replicates (2 ducks/replicate) per treatment. Data were expressed as mean ± SD. Values within the same gene or time with no common lowercase letters (a−b) differ significantly (*P* < 0.05).

The effect of photoperiod on the relative mRNA expressions of reproduction-related genes in the hypothalamus and serum level of prolactin is detailed in Figures 1A and 1B. Compared with 12L:12D, 16L:8D and 18L:6D treatments upregulated the mRNA expressions of GnRH, and the higher mRNA expressions of GnIH and vasoactive intestinal peptide were observed in 20L:4D treatment, accompanied with the higher prolactin level in serum (0:00). Besides, the numerically highest values of serum prolactin level occurred consistently in 20L:4D treatment at all the 4 time points.

## Discussion

The potential benefits of photoperiod on poultry productive performance have been extensively reported, such as increasing egg mass of broiler breeders (Lewis and Gous, 2006), laying hens (Geng et al., 2014), and turkey breeders (Siopes, 2007), as well as promoting feed intake of turkeys (Schwean-Lardner et al., 2012). Similar results were observed in our present study that 16- to 20-hour photoperiods increased the egg mass of laying ducks from 37 to 44 wk of age, which is consistent with the report that 16-hour photoperiod conferred Beijing You Chicken the higher egg mass compared with that of 14-hour photoperiod during 44 to 57 wk of age (Geng et al., 2018). The higher egg mass may be attributed to the improvement of egg production. In fact, higher egg production was simultaneously observed in 16- to 20-hour photoperiod treatments in this research, without significant change in egg weight. Consistently, Molino et al. (2015) found that increase in photoperiods could significantly enhance egg production of Japanese quails during laying period. These results may be ascribed to the positive effect of prolonging photoperiod on serum reproductive hormone level and ovarian follicle development (Cui et al., 2019a). Besides, the higher ADFI was always associated with more egg mass (Marume et al., 2020), which was also verified by our result that consistent increases were observed in egg mass and ADFI, accompanied with no change in feed conversion ratio. The higher ADFI driving by appropriate photoperiod (Schwean-Lardner et al., 2012) may be owing to the combination of better mobility and longer feeding (Schwean-Lardner et al., 2013, 2014; Cui et al., 2019b). The higher egg mass, egg production, and ADFI (1–4, 5–8, and 1–8 wk of experiment) implied 16- to 20-hour photoperiods were suitable for productive performance of laying ducks during 37 to 44 wk of age.

Laying performance of birds has a close link with the development of reproductive organs, including the oviduct, ovary, and ovarian stroma (Chen et al., 2007; Cui et al., 2019a). The photoperiod may promote the development of the reproductive organ, serving as a driving force, whereas an inhibitory effect occurs with superabundant photoperiod (Chen et al., 2007; Cui et al., 2019a). In our study, initial stroma weight, bare stroma weight, and percentage increased quadratically in response to the increment in photoperiod, and significantly higher initial and bare stroma weight occurred in 16L:8D and 18L:6D treatments. These findings may imply that a photoperiod of 16 to 18 h was suitable for reproductive organ development of laying ducks. Next, the photoperiods' effect was further investigated by quadratic regression analysis. The reliable equation for bare stroma weight stood out: y = −0.02714 × ^2^ + 0.89907x – 2.70710, *R*^*2*^ = 0.5096, and 16.56 was the optimal photoperiod, obtaining from calculation. Therefore, a photoperiod of 16.56 h was supposed to be the most appropriate for the reproductive organ development of laying ducks.

The response of reproductive organ development to photoperiod was always consistent with that of ovarian follicle development (Cui et al., 2019a). In the present study, total LWF number and weight increased quadratically with the increasing photoperiods, and the highest values of them occurred in16L:8D and 18L:6D treatments. The higher weight and larger number mean the better development of follicles and thus a better laying performance (Bulbul et al., 2015; Long et al., 2017), which was consistent with the result mentioned previously that 16L:8D and 18L:6D treatments performed better in egg production and egg mass. The optimal photoperiod for follicle development was pursued through quadratic regression analysis, and the reliable equation was obtained for total LWF weight: y = −0.0116 × ^2^ + 0.37788x – 2.01352, *R*^*2*^ = 0.5071. Based on this equation, the optimal photoperiod was calculated as 16.93 h/day. Taking the development of the reproductive organ and ovarian follicle into consideration, a 16.56- to 16.93-hour photoperiod could be appropriate for laying ducks.

The development of ovarian follicles and reproductive organs is mainly regulated by the HPG axis (Cui et al., 2019a), through the secretion and release of various reproductive hormones, including estradiol, FSH, LH and progesterone. Follicle-stimulating hormone and LH, the primary gonadotropins, could not only promote oviduct, ovary, and stroma development (Long et al., 2017) but also play significant roles in the process of follicular development and ovulation (Cui et al., 2019a). Follicle-stimulating hormone acts as the main driving force for the development and maturation of small follicles, especially for F6 to F3 follicles, SYF, and LWF (Hernandez and Bahr, 2003). In contrast, the granulosa layer of the larger POF is the main target of LH (Zhang et al., 1997). In fact, serum hormone level has been supposed to be a sensitive indicator for performance of laying birds (Mohammadi and Ansari-Pirsaraei, 2013). In our present study, the higher levels of FSH occurred in ≥16-hour photoperiods, and the higher values of LH were observed in 16L:8D and 18L:6D, which was consistent with the better development of reproductive organ and follicles in these treatments. These may be responsible for the positive effects of these photoperiods on the development of reproductive organs and ovarian follicles (Cui et al., 2019a). Moreover, FSH and LH were reported to promote the secretion and release of progesterone and estradiol in thecal and granulosa cells of ovarian follicles (Hrabia et al., 2004). Consistently, the higher serum levels of estradiol and progesterone were observed in photoperiods ≥16 h in this present study, which could indirectly suggest the better ovarian follicles development in these treatments. Progesterone acts as a driving force in the ovulation of hens (Nakada et al., 1994), which may be attributed to the activation of the progesterone receptor in granulosa cells of preovulatory follicles (Natraj and Richards, 1993). Estradiol, the major estrogen, promotes the proliferation of follicular granulosa cells and the release of gonadotropins in pituitary and amplifies the positive feedback effects of progesterone on the HPG axis (Ing and Tornesi, 1997).

The higher values of these 4 hormones occurred in 16L:8D and 18L:6D treatments, and 3 of them were also observed in 20L:4D, which was consistent with the responses of ovarian morphology and laying performance to photoperiod. Thus, the positive effects of photoperiod on laying performance and reproductive organ and ovarian follicle development could be attributed to the increase in levels of reproductive hormones. The rise in serum FSH and LH probably led to the increase in the secretion of estradiol and progesterone, while the latter may stimulate the release of the former in turn. The biological effects of estradiol, FSH, LH, and progesterone on target cells can be carried out through their receptors (Wang et al., 2016). Therefore, the mRNA expressions of aforementioned hormone receptors gene were further detected. In the present research, mRNA expressions of estrogen, FSH, LH, and progesterone receptors were observed in F1, F2, F3, SYF, and LWF samples, which meant that these 4 hormones could act directly on follicles development, similar to the previous studies (Hrabia et al., 2004, 2008). The higher mRNA expressions of these 4 hormone receptors were observed in 16L:8D and 18L:6D treatments, and 3 of them occurred also in 20L:4D treatment, which was consistent with the responses of serum reproductive hormone to photoperiod. These findings may be ascribed to the increment in hormones inducing the expression of their receptors (Ing and Tornesi, 1997). Taking serum reproductive hormones and their receptors gene mRNA expressions into consideration, 16- to 20-hour photoperiods may be appropriate, among them 16- to 18-hour photoperiods performed better.

The changes happened in serum levels of reproductive hormones can upstream trace to the effect of photoperiod on GnRH neurons in the hypothalamus (Sharp, 1993). Hence, the release of GnRH and regulation of GnRH neurons were further investigated in this study. The higher mRNA expressions of GnRH gene were observed in 16- and 18-hour photoperiods in the hypothalamus of laying ducks at 310 d of age, consistent with the better development of reproductive organs and ovarian follicles in these 2 treatments. These findings implied that a 16- to 18-hour photoperiod was suitable and it could stimulate the HPG axis and raise the secretion and release of GnRH. In poultry, the secretion and release of GnRH could be regulated by GnIH (Banerjee and Chaturvedi, 2017), Dio2/Dio3 system (Perfito et al., 2015), or prolactin (Fox et al., 1987). In this research, there was no significant difference in the mRNA expressions of Dio2 and Dio3, while that of GnIH were upregulated in 20-hour photoperiod accompanied with the higher prolactin level in serum. Gonadotropin-inhibitory hormone, a peptide hormone separated from the quail brain (Tsutsui et al., 2000), could probably inhibit the synthesis and secretion of GnRH (Ubuka et al., 2008). Prolactin was reported to suppress the pituitary responsiveness to GnRH in the birds (Sharp and Blache, 2003). Our results showed that 12- to 20-hour photoperiods did not impact the access of Dio2/Dio3 system on GnRH neurons, while 20-hour photoperiod could suppress the expression of GnRH through raising the expression of GnIH and the level of prolactin. The increase in serum level of prolactin may be attributed to the upregulation of mRNA expression of vasoactive intestinal peptide gene, which acts as the activator of prolactin (Bhatt et al., 2003). For laying ducks, the mRNA expression of GnRH was upregulated with 16- and 18-hour photoperiods, while it was downregulated when photoperiod reached 20 h because of the inhibiting effects of GnIH and serum prolactin.

In conclusion, an increment in photoperiod improved productive performance of laying ducks in a linear and quadratic manner. Moreover, photoperiod quadratically promoted the development of reproductive organs and follicles, with 16.56 to 16.93 h as the optimal photoperiod. Besides, the higher levels of serum estradiol, FSH, LH, and progesterone accompanied with their receptors mRNA expressions were observed in ≥16-hour photoperiods, and the overall optimal values occurred in 16- to 18-hour photoperiod. These results could be ascribed to GnRH expression increasing in these 2 photoperiods and being suppressed by GnIH and prolactin when photoperiod reached 20 h. Thus, 16.56 to 16.93 h was the most suitable photoperiod for productive performance, ovarian morphology development, and reproductive hormone secretion of laying ducks.
